# Additional jump interval training as a form of warm-up on enhancing aerobic capacity, muscular strength, and power in female dancers: a cohort study

**DOI:** 10.3389/fphys.2025.1504468

**Published:** 2025-07-01

**Authors:** Qianqian Sun, Mingzhu Wu, Yike Ni

**Affiliations:** ^1^ Department of Public Teaching, Guangzhou Panyu Polytechnic, Guangzhou, China; ^2^ Gdansk University of Physical Education and Sport, Gdańsk, Poland; ^3^ Guangzhou Sport University, Guangzhou, China

**Keywords:** physical exercise, physical fitness, high-intensity interval training, microdosing, youth

## Abstract

**Objectives:**

This study aimed to compare the effects of jump interval training (JIT) as a supplement to a warm-up with a control group that continued their regular warm-up routine, on the development of aerobic capacity, maximal isometric strength, and vertical jump power in female dancers over a 6-month period.

**Methods:**

Fifty-five female sport dancers (aged 16.2 ± 0.9 years) were monitored throughout the 6 months, with evaluations conducted at baseline, 3 months, and 6 months. Aerobic capacity was assessed using the multistage fitness test (MFT), while muscular assessments included squat jump (SJ), countermovement jump (CMJ), and isometric mid-thigh pull test (IMTP) measured on force platforms. The participants were analyzed into two cohorts: one group adhered to the JIT warm-up, which involved three sets of 30-s bilateral squat jumps during the potentiation phase, while the control group (TWU) performed ballistic dance movements emphasizing explosive techniques, such as rapid leaps and forceful limb movements, in the same phase.

**Results:**

After 6-months, significant differences were observed between groups in IMTP (*F* = 8.702; *p* = 0.005; *d* = 0.796, moderate effect size), SJ (*F* = 5.454; *p* = 0.023; *d* = 0.637, moderate effect size), CMJ (*F* = 5.921; *p* = 0.018; *d* = 0.659, moderate effect size), and MFT (*F* = 52.370; *p* < 0.001; *d* = 1.950, large effect size).

**Conclusion:**

The study concludes that incorporating JIT exercises into warm-up routines significantly enhances aerobic capacity and muscular performance over 6 months, with early improvements in aerobic capacity and noticeable benefits in muscular performance such as SJ peak force, CMJ peak power, and IMTP peak force after three and 6 months. This study contributes to the field as one of the few that demonstrates the relevance and practicality of integrating microdosing (i.e., small but intensive JIT sessions throughout the week) into dance warm-up routines.

## Introduction

Female dancers face physical demands that require exceptional cardiovascular and muscular capabilities. The complex and intense choreography and dynamic movements of dance routines require high levels of aerobic capacity, enabling sustained performance without compromising technique or expression (Liiv et al., 2014). Studies suggests that dancers with enhanced aerobic capacities exhibit superior endurance and recovery, crucial for maintaining peak performance throughout rehearsals and performances (Smol and Fredyk, 2012; Angioi et al., 2009). Additionally, high-intensity interval training is recommended for dancers seeking to improve their aerobic capacity to meet these demands (Rodrigues-Krause et al., 2015). Alongside cardiovascular endurance, muscular strength and power are also essential for dance performance. These attributes are critical for executing explosive movements and achieving the agility required in dance (Uzunovic et al., 2010). Dancers with well-developed muscular strength and power demonstrate improved balance, and stability, which are indispensable for mastering dance movements (Long et al., 2021).

As part of dance training routines, incorporating warm-up exercises is of particular importance. For example, a study examining the effects of different stretching warm-up strategies in dancers found that combined stretching significantly improved balance, vertical jump height, and both pre-stretch and post-stretch range of motion (Morrin and Redding, 2013). When comparing stretching to dynamic warm-ups, a study conducted on dancers revealed that both types of warm-up routines led to similar improvements in range of motion (ROM) and motor abilities; however, it was noted that dynamic warm-ups slightly enhanced motor abilities more than stretching (Zaggelidou et al., 2023). In addition to performance benefits, neuromuscular warm-ups have been found to potentially offer protection against overuse injuries in dancers, according to findings from a 2-year prospective cohort study (Kaufmann et al., 2022).

Warm-up strategies can be optimized by incorporating microdosing of specific training interventions that can impact long-term physical fitness adaptations. For example, in physical education classes, incorporating additional core stability exercises into warm-ups enhanced muscular endurance, movement capability, flexibility, and balance (Chang et al., 2020). In wrestlers, it was observed that incorporating a dynamic stretching warm-up intervention over a 4-week period led to positive long-term adaptations in power, strength, muscular endurance, anaerobic capacity, and agility performance (Herman and Smith, 2008).

While warm-up strategies vary, it is widely accepted that the warm-up can be structured into four phases, following the RAMP strategy (Raise, Activate, Mobilize, Potentiate) (Jeffreys, 2017). The RAMP strategy consists of four phases: Raise—gradually increasing heart rate and body temperature through light aerobic activity; Activate—engaging specific muscles with dynamic movements to prepare them for the demands of the activity; Mobilize—improving joint range of motion through dynamic stretching; and Potentiate—performing sport-specific, high-intensity drills to optimize performance before the main activity (Jeffreys, 2017). During the Potentiate phase, the goal is to increase physiological and neuromuscular stress (McGowan et al., 2015), which can be leveraged to introduce microdosing of a training intervention. This approach may not only impact the immediate response to the session but also lead to long-term adaptations, similar to those seen with ballistic movements, for instance (Johnson et al., 2019).

Considering that dancers may benefit from enhancing aerobic capacity and muscular adaptations, strategies that target both aspects can be particularly promising for long-term improvements in their performance. One potentially beneficial approach is jumping interval training (JIT) (Kramer et al., 2019; Ache-Dias et al., 2016), which involves short bursts of high-intensity jumping exercises interspersed with brief periods of rest or lower-intensity activity. This training modality utilizes jumps as a primary exercise component and follows to the principles of high-intensity interval training, aiming to effectively engage muscles through dynamic and intense movements (Kramer et al., 2019; Ache-Dias et al., 2016). Scientific evidence supports that JIT can enhance cardiovascular fitness due to its high-intensity heart rate stimulus and the increased physiological demands that promote cardiovascular adaptations (Ma et al., 2024). Additionally, JIT not only improves aerobic capacity but also stimulates neuromuscular adaptations crucial for enhancing muscular strength and power (Ma et al., 2024). This is likely due to the repeated bouts of multiple jumps, which require reactive strength and contribute to improved muscular endurance.

Despite the importance of enhancing aerobic capacity and muscular strength and power, such as maximal strength or vertical jump, research investigating the impact of training on these characteristics in dancers is limited (Sanders et al., 2020; Roussel et al., 2014). Furthermore, there is a lack of research on how warm-up protocols, specifically incorporating small yet consistent doses of interventions like JIT, could be relevant for dancers. This type of analysis could identify approaches that minimize intervention time and optimize strategies to enhance physical fitness without disrupting the core content of dance sessions, which typically focus more on technical elements and choreography. Despite its potential, no study to date has tested JIT as a complementary approach for dancers, specifically as a supplement to warm-up routines, creating a gap that can be addressed.

Considering the significance and innovative approach of this study, the aim was to compare the effects of JIT as a supplement to a warm-up with a control group of dancers who continued their regular warm-up routine, on the development of aerobic capacity (measured by the multistage fitness test, MFT), muscular strength (assessed by the isometric mid-thigh pull test, IMTP), and power (measured by squat jump, SJ, and countermovement jump, CMJ) in female dancers over a 6-month period.

## Methods

### Study design

This study consisted in a prospective cohort design to monitor dancers’ development of aerobic capacity, muscular strength, and power over a period of 6 months, with assessments conducted three times (baseline, 3-month and 6-month). The participants were classified into two cohorts: one group (JIT) incorporated a JIT micro-intervention within their warm-up protocol, while the other group (TWU) continued with their regular warm-up routines. Participants were enrolled based on their willingness to participate, with those expressing interest in the warm-up supplemented with JIT included in that group, while those who did not opt for JIT continued with their regular routines. The study followed the STROBE guidelines for reporting cohort studies (Cuschieri, 2019).

### Setting

The study lasted 6 months, with evaluations consistently conducted under the same conditions (i.e., during the first training session of the week, in the afternoon, and following the same sequence). Assessments took place in three dance academies, with evaluations at baseline, 3 months, and 6 months all performed at the same location to ensure replicability of the conditions. The dance classes offered included ballet, folk dance, and hip hop. All participants were recreational dancers with an intermediate skill level. The recruitment process took place over 3 weeks prior to the baseline assessment. It involved reaching out to the dance academy directors and inviting participants and their parents to join the study.

### Participants

The inclusion criteria for participants were as follows: (i) being female; (ii) aged between 15 and 18 years; (iii) not having injuries or severe illnesses that could compromise adherence to training sessions or cause them to miss evaluation periods; (iv) participating in at least 85% of the total training sessions scheduled by their academies; (v) not participating in any other training programs besides dance at their academies; and (vi) having more than 2 years of dancing experience. Exclusion criteria included: (i) participants who changed their cohort participation (e.g., switched from JIT to control or *vice versa*); (ii) missing any of the evaluations (i.e., instances where participants did not complete one or more of the scheduled assessments due to personal reasons, scheduling conflicts, or other unforeseen circumstances); (iii) declaring participation in additional training programs outside the regular sessions conducted by the academies; (iv) missing more than 15% of the overall training sessions scheduled during the observational study period.

After recruiting participants by directly contacting dance academies and reaching out to volunteers and their legal guardians through public announcements, we found a total of 62 participants. Based on the eligibility criteria, 5 participants were excluded before the baseline assessment: 3 were <14 years old and 2 had lower limb injuries. No participants were excluded due to attendance criteria or missing evaluations. During the follow-up, two additional participants were excluded due to leaving the dance academy for personal reasons. Consequently, 55 participants were successfully monitored from the baseline to the final evaluation (6-month mark). Among these participants, 26 voluntarily adhered to the JIT warm-up and continued with this option throughout the observation period, while the remaining 29 continued their traditional warm-up routines.

Overall (n = 55), the participants had average measurements of 16.2 ± 0.9 years, 1.59 ± 0.03 m in height, 50.3 ± 2.3 kg in body mass, and 19.8 ± 0.6 kg/m^2^ in body mass index. Dancers adhering to JIT (n = 26) had average measurements of 16.2 ± 0.9 years, 1.60 ± 0.03 m, 50.7 ± 2.0 kg, and 19.9 ± 0.7 kg/m^2^. Dancers in TWU (n = 29) had average measurements of 16.1 ± 0.8 years, 1.59 ± 0.03 m, 49.9 ± 2.5 kg, and 19.8 ± 0.5 kg/m^2^. All sport dancers trained two to three times per week, with each session lasting between 80 and 110 min. They were classified as sport dancers based on the Participants Classification Framework (McKay et al., 2022), which defines them as trained/developmental dancers who attend classes three times a week and occasionally participate in competitions. The participants were at an intermediate level, having some prior dance experience but still in the process of refining their skills. Specifically, all participants had a minimum of 2 years of practice and had been introduced to the core techniques of the dance style. However, most were not competitive dancers and had not yet reached a level of proficiency free from technical errors. As such, all participants were still actively developing their technical skills in classes focused on improvement.

The Ethical Evaluation of Human Experiment of Guangzhou Sport University granted approval for this study (2024LCLL-92). Participants and their legal guardians received information regarding the study’s procedures, potential benefits, risks, and the voluntary nature of their involvement. Informed consent was obtained from the legal guardians of participants under 18 years old, with each guardian signing a consent form to confirm voluntary participation. For participants aged 18 and over, they themselves signed the informed consent form. The research adhered to the ethical standards set forth in the Declaration of Helsinki.

### Variables

The participants were classified into two cohorts: those who adhered to JIT warm-up and those who maintained TWU. This classification served as the independent variable in the study. As background, dance academy instructors had proposed a new warm-up routine (including JIT) to replace the traditional phase of ballistic technical elements that typically followed mobilization exercises. Under this new approach, the warm-up consisted of three phases in RAMP, with identical structure across all participants, except for the potentiation phase (fourth phase), which varied (JIT vs. control).

Although the warm-up content could vary over time, it was standardized across all participants except for the final phase. In the potentiation phase, participants adhering to JIT performed three sets of 30-s bilateral repeated squat jumps at a pace of 1.0 jump per second, interspersed with 30 s of rest. This routine was performed during all dance sessions (approximately 2–3 times per week). Conversely, participants in the TWU group maintained a potentiation phase involving ballistic dance movements (e.g., leaping Jump; saut de basque; Popping; fan dance leaps) emphasizing explosive techniques, such as rapid leaps and forceful limb movements. Slight variances were observed across the different academies, and to ensure consistency, an observational record was kept, showing similar trends in the selected movements.

The dependent variables in this study included peak vertical force in the isometric mid-thigh pull test (IMTP), peak force in the squat jump test (SJ), peak power in the countermovement jump test (CMJ), and total distance covered in the multistage fitness test (MFT). The relevance of studying these variables lies in their ability to assess key components of physical performance that are critical for dancers. The IMTP and SJ measure strength, which is fundamental for stability, control, and power in dance movements (Angioi et al., 2009). The CMJ reflects explosive movement ability, essential for jumps and rapid directional changes in dance (Blanco et al., 2021). Finally, the MFT provides insight into aerobic capacity, crucial for endurance during extended dance sessions (Redding and Wyon, 2003).

### Procedures of assessment

The female dancer underwent three assessments: baseline, after three and 6 months. The evaluations were conducted under consistent conditions, specifically during the first training session of the week, following 48 h of rest. The same assessors evaluated all the athletes across all dance academies and time points, ensuring consistent evaluation conditions. The anthropometric measurements (height and body mass, collected only at baseline to characterize the participants) and muscular assessments took place in the afternoons within a room maintained at 22°C with a relative humidity of 50%. The aerobic capacity test was conducted into an indoor facility.

On evaluation day, participants first provided demographic information (age, years of experience, type of dance) and underwent anthropometric measurements (height and body mass). They then followed a standardized warm-up protocol, which included 5 min of moderate intensity jogging into the indoor facility, followed by dynamic stretching exercises for the upper limbs (7 min) and lower limbs (7 min). After dynamic stretching, participants performed reactive jumps and landing movements to prepare for the evaluations.

After 3 min of completing the warm-up, all participants followed the same sequence of evaluations: (i) isometric mid-thigh pull test (IMTP), (ii) squat jump test (SJT), (iii) countermovement jump test (CMJ), and (iv) multistage fitness test (MFT). Each muscular test included two trials interspaced by 3 min of rest. Before the recorded trials, participants completed a familiarization trial to ensure they understood the correct movements for each test. A 5-minute rest period was provided between each test.

### Isometric mid-thigh pull test

For the IMTP assessment, athletes adopted a standardized power-pulling stance as recommended in previous studies, with knee angles set between 130° and 140° and hip angles at 145° (Comfort et al., 2015). The bar height was adjusted to half the distance between the greater trochanter and the lateral epicondyle of the knee, using a goniometer for precise alignment within acceptable ranges (Merrigan et al., 2020). The bar was secured firmly in place to eliminate any slack. Wrist straps were used to secure the athletes’ hands to the bar, standardizing grip force and ensuring consistency in the measurements (Merrigan et al., 2020). Participants were instructed to apply maximum force on the bar for 3 seconds, focusing on explosive upward movement. The tests were conducted on a force platform (Quattro Jump, Kistler, Winterthur, Switzerland), with the average peak vertical force calculated from two trials used for subsequent data analysis.

### Squat jump test

Participants performed an unloaded squat jump, selecting their preferred foot position. This position was measured and standardized to ensure consistency across all tests, in accordance with previous protocols (Sheppard and Doyle, 2008). Starting from a squat position at a self-determined comfortable depth with hands on their hips, they were instructed to jump with maximal effort. They aimed to extend their knees powerfully and land smoothly on the force platform with both feet simultaneously. Peak force (N) was recorded for each trial, and the average was used for subsequent data analysis.

### Countermovement jump test procedures

Participants performed the traditional CMJ test, beginning from a standing position on the force platform, in accordance with previous protocols (Owen et al., 2014). Following instructions, they initiated the jump by swiftly descending into a comfortable partial squat through hip and knee flexion. This was immediately followed by an explosive extension of the hips, knees, and ankles to achieve maximum vertical height, with hands kept on the hips throughout the movement. Participants were instructed to keep their knees extended while in the air and to land smoothly on both feet simultaneously. Peak power (W/kg) were recorded for each trial, and the averages were used for subsequent data analysis.

### Multistage fitness test

The MFT, also known as the 20 m shuttle run test, was implemented based on Leger’s original work (Léger et al., 1988). The test comprised 23 levels, with each level lasting approximately 1 minute. The starting speed was 8.5 km/h and increased by 0.5 km/h at each subsequent level. During the test, a single beep indicated the end of each shuttle, while three simultaneous beeps indicated the start of the next level. If an athlete failed to reach the opposite “turn-around” line before the beep, they were issued one failed attempt. If they failed two consecutive attempts, they were withdrawn from the test, and their score was recorded as final. However, if the individual reached the line before the second consecutive beep, their failed attempts were reset. The total distance completed (m) before participants gave up or were excluded was considered the main outcome of the test.

### Study size

Before starting the research, the sample size was calculated using G*Power software. To achieve a power of 0.85 and an effect size (f) of 0.2 for two groups measured at three different time points, a total of 48 participants was recommended for the repeated measures ANOVA.

### Statistical methods

To compare the two cohorts (JIT and TWU) across three time points, a mixed ANOVA was employed, allowing for the examination of interactions between groups and evaluation moments. The assumptions of normality and homogeneity were verified using the Kolmogorov-Smirnov test (p > 0.05) and Levene’s test (p > 0.05), respectively. Post hoc analyses were performed using the Bonferroni test to compare pairs of time points (baseline, 3 months, and 6 months), with partial eta squared employed to measure the effect size. Moreover, the standardized effect size (Cohen’s d) was used for pairwise comparisons, following the thresholds (Hopkins et al., 2009): 0.0–0.2 for trivial ES, 0.2–0.6 for small ES, 0.6–1.2 for moderate ES, and >1.2 for large ES. All statistical analyses were performed with IBM SPSS Statistics (Version 27.0, IBM Corp., Armonk, NY), with significance set at p < 0.05.

## Results

**FIGURE 1:**
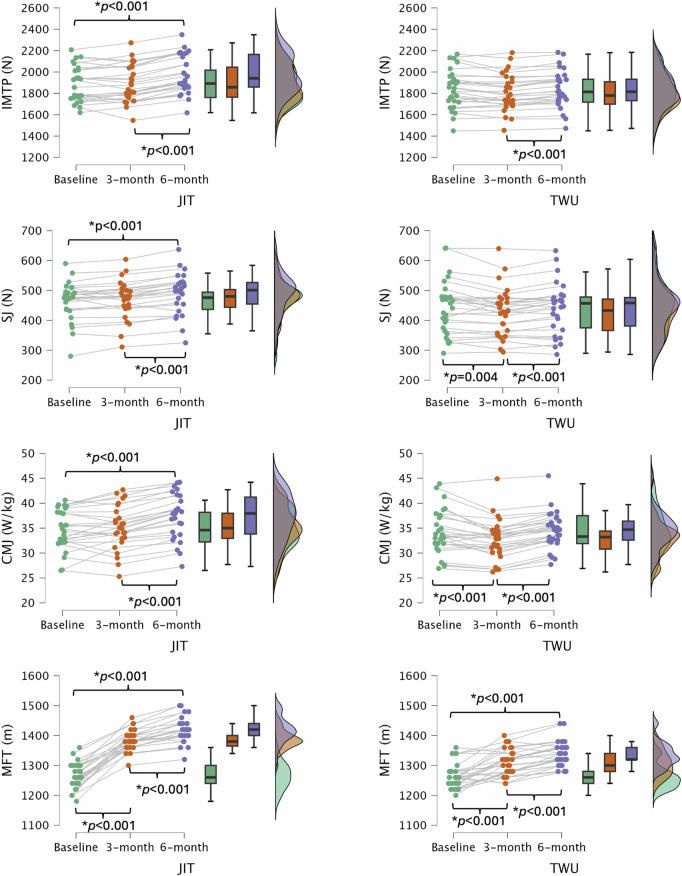
Variations in physical fitness outcomes for both groups. JIT, jump interval training; TWU, standard warm-up; IMTP, isometric mid-thigh pull test; SJ, squat jump; CMJ, countermovement jump; MTF, multistage fitness test. *: significantly different (p < 0.05).

Table 1 presents the descriptive statistics for muscular performance outcomes and aerobic capacity across the groups. Figure 1 illustrates the variations in physical fitness outcomes between the two groups.

**TABLE 1:** Descriptive statistics (mean ± standard deviation) of the muscular performance outcomes and aerobic capacity between groups.

	JIT (n = 26)	TWU (n = 29)	JIT vs. TWU Mean difference	JIT vs. TWU *p*-value	JIT vs. TWU Effect size (d)
IMTP (N)					
Baseline	1887.4 ± 166.8^£^	1830.2 ± 179.9	57.2	*p* = 0.228	0.330
3-month	1891.8 ± 176.9^£^	1807.6 ± 169.8^£^	84.2	*p* = 0.077	0.486
6-month	1980.5 ± 181.8^#,$^	1839.3 ± 173.1^$^	141.2	*p = 0.005	0.796
6-month vs. baseline (d)	0.534, small ES	0.052, trivial ES			
SJ (N)					
Baseline	464.7 ± 64.7^£^	443.7 ± 89.0^$^	21.0	*p* = 0.326	0.273
3-month	470.5 ± 64.3^£^	430.4 ± 78.7^#^	40.2*	*p = 0.044	0.561
6-month	490.4 ± 68.8^#,$^	441.6 ± 84.3	48.8*	*p = 0.023	0.637
6-month vs. baseline (d)	0.385, small ES	0.024, trivial ES			
CMJ (W/kg)					
Baseline	34.6 ± 4.0^£^	34.2 ± 4.4^$^	0.38	*p* = 0.741	0.095
3-month	35.1 ± 4.5^£^	32.8 ± 3.9^#^	2.36*	*p = 0.041	0.548
6-month	37.3 ± 4.6^#,$^	34.6 ± 3.6	2.71*	*p = 0.018	0.659
6-month – baseline (d)	0.628, moderate ES	0.100, trivial ES			
MFT (m)					
Baseline	1,265.4 ± 43.7^$,£^	1,261.4 ± 41.4^$,£^	4.0	*p* = 0.728	0.094
3-month	1,385.4 ± 34.2^#,£^	1,308.3 ± 43.6^#,£^	77.1*	*p < 0.001	1.982
6-month	1,419.2 ± 42.9^#,$^	1,336.6 ± 41.8^#,$^	82.7*	*p < 0.001	1.950
6-month vs. baseline (d)	3.552, large ES	1.808, large ES			

JIT, jump interval training; TWU, standard warm-up; IMTP, isometric mid-thigh pull test; SJ, squat jump; CMJ, countermovement jump; MTF, multistage fitness test; ES, effect size; *significantly different between groups (p < 0.05); ^#^: significantly different from baseline evaluation (p < 0.05); ^$^: significantly different from 3-month evaluation (p < 0.05); ^£^: significantly different from 6-month evaluation (p < 0.05).

Significant interactions were found between groups and the time in regards the IMTP (*F* = 27.750; *p* < 0.001; partial eta squared = 0.344), SJ (*F* = 15.777; *p* < 0.001; partial eta squared = 0.229), and MFT (*F* = 42.184; *p* < 0.001; partial eta squared = 0.443).

The within-JIT analysis revealed significantly higher IMTP values after 6 months compared to baseline (mean difference: 93.2 N; *p* < 0.001) and after 3 months (mean difference: 88.7 N; *p* < 0.001). Similarly, within-TWU, higher IMTP values were observed after 6 months compared to the 3-month evaluation (mean difference: 31.7 N; *p* < 0.001).

Regarding the SJ, significantly greater values were observed after 6 months compared to baseline (mean difference: 25.7 N; *p* < 0.001) and after 3 months (mean difference: 19.9 N; *p* < 0.001) in the JIT group. Additionally, in the TWU group, the 3-month evaluation showed significantly lower SJ values compared to baseline (mean difference: 13.3 N; *p* = 0.004) and after 6 months (mean difference: 11.2 N; *p* < 0.001).

The within-JIT analysis revealed significantly higher CMJ values after 6 months compared to baseline (mean difference: 2.7 W/kg; *p* < 0.001) and after 3 months (mean difference: 2.2 W/kg; *p* < 0.001). Similarly, within the TWU group, higher CMJ values were observed after 6 months compared to the 3-month evaluation (mean difference: 1.8 W/kg; *p* < 0.001), and baseline values were also significantly higher than those at the 3-month assessment point (mean difference: 1.4 W/kg; *p* < 0.001).

Regarding the MFT, significantly greater values were observed after 6 months compared to baseline (mean difference: 153.8 m; *p* < 0.001) and after 3 months (mean difference: 33.8 m; *p* < 0.001) in the JIT group. Additionally, in the TWU group, the baseline evaluation showed significantly lower MFT values compared to after 3 months (mean difference: 46.9 m; *p* < 0.001) and after 6 months (mean difference: 75.2 m; *p* < 0.001), and after 6 months values were also significantly higher than those at the 3-month assessment point (mean difference: 28.3 m; *p* < 0.001).

In the baseline, no significant differences were observed between groups in IMTP (*F* = 1.485; *p* = 0.228), SJ (*F* = 0.984; *p* = 0.326), CMJ (*F* = 17.930; *p* < 0.001), CMJ (*F* = 0.110; *p* = 0.741), and MFT (*F* = 0.122; *p* = 0.728).

In the 3-month evaluation significant differences were found between groups in SJ (*F* = 4.236; *p* = 0.044), CMJ (*F* = 4.371; *p* = 0.041), and MFT (*F* = 52.390; *p* < 0.001), although no significant differences were observed between groups in IMTP (*F* = 3.243; *p* = 0.077).

After 6-months, significant differences were observed between groups in IMTP (*F* = 8.702; *p* = 0.005), SJ (*F* = 5.454; *p* = 0.023), CMJ (*F* = 5.921; *p* = 0.018), and MFT (*F* = 52.370; *p* < 0.001).

## Discussion

Our cohort study found that, after the third month, the increases in aerobic capacity, SJ peak force, and CMJ peak power were significantly greater in the JIT cohort compared to the TWU cohort. Additionally, at the 6-month evaluation, the JIT cohort exhibited significantly greater maximal strength in the IMTP compared to the TWU cohort. The significant differences in the other outcomes observed at the third month persisted similarly through the 6-month period.

It was observed that JIT significantly contributed to enhancing aerobic capacity, resulting in consistent improvements that became significantly different from TWU after the 3-month evaluation. Our results align with a previous study which found that 3 weekly sessions over 6 weeks of JIT significantly enhanced absolute maximal aerobic capacity, though not the relative measure (Venegas-Carro et al., 2023). Additionally, our findings are consistent with a study conducted on gymnasts, which revealed significant improvements in MFT after 8 weeks in the JIT group (Ma et al., 2024).

As a form of high-intensity interval training that uses jumping as the modality, this type of exercise can induce cardiovascular improvements, including increased stroke volume and cardiac output (Engel et al., 2018). The repetitive and intense nature of jumping interval training (JIT), which often involves spending a significant amount of time above 90% of maximal oxygen uptake (Kramer et al., 2019), likely stimulates greater capillary density within skeletal muscles, enhancing oxygen delivery (Jacobs et al., 2013). It is also hypothesized that, similar to other high-intensity interval training methods, small dose JIT may promote mitochondrial biogenesis (Little et al., 2010), enhancing mitochondrial density and efficiency and improving oxidative phosphorylation. The use of jumping is particularly relevant, as it aligns with the types of movements promoted by the dance styles analyzed, thereby increasing the specificity of the training while providing an effective and time-efficient aerobic stimulus.

Our study also found that after a 3-month evaluation, the cohorts showed significant differences in squat jump (SJ) peak force and countermovement jump (CMJ) peak power. These results align with previous studies, such as one that used JIT three times a week over 4 weeks in recreational runners, which found increases in jump height and power output of the CMJ (Ache-Dias et al., 2016). Similar improvements in CMJ height were observed after 8 weeks of JIT training in female gymnasts (Ma et al., 2024).

The powerful nature of jumping exercises may stimulate a higher degree of motor unit recruitment and synchronization (Chimera et al., 2004). The repetitive, high-intensity jumps likely enhance neuromuscular efficiency by facilitating more rapid and efficient motor unit firing patterns, which are crucial for generating peak force and power (Behrens et al., 2016). Additionally, JIT may promote adaptations in the muscle-tendon unit due to the repetitive jumps and landings, increasing tendon stiffness (Fouré et al., 2010), which allows for more effective storage and release of elastic energy during dynamic movements. Enhanced intramuscular coordination and the activation of fast-twitch muscle fibers could also explain the greater force production and power output (Komi, 2003). These adaptations may be less prominent in technical ballistic dance movements, which often involve more submaximal, less explosive muscle contractions and do not stimulate the same level of neuromuscular adaptation necessary for maximal power and force production in jumps.

Interestingly, after 6 months, evaluations revealed that the cohorts also became significantly different in terms of IMTP peak force, with those adhering to JIT showing greater force. These results align with previous studies, such as one that used JIT three times a week over 4 weeks in recreational runners, which found increases in knee extensor strength during isometric contraction (Ache-Dias et al., 2016). Another study observed significant improvements in lower limb strength after a 6-week JIT intervention (Venegas-Carro et al., 2023).

The potentially increased firing rates of motor units and enhanced recruitment of fast-twitch muscle fibers (Komi, 2003) following JIT may explain the ability to generate high force outputs in isometric contractions like the IMTP. JIT possibly stimulated adaptations in the central nervous system, improving the capacity to generate force rapidly and efficiently (Davies et al., 2015). These adaptations may include heightened motor cortex excitability and enhanced motor unit synchronization (Ramirez-Campillo et al., 2021), crucial for maximizing peak force production during isometric contractions. In contrast, technical ballistic dance movements typically entail lower-force, rhythmic muscle actions that do not induce the same degree of neuromuscular adaptation required for high-force isometric contractions.

While our study shows interesting findings regarding the benefits of JIT integrated in warm-up on aerobic capacity and muscular performance, some limitations warrant consideration. Firstly, our cohort study design limits causal inference due to the absence of randomization, potentially introducing selection bias. Moreover, integrating different dance styles may have influenced the results, as each style imposes a different workload due to its unique routines and movements. Additionally, the study duration of 6 months may not capture longer-term effects or potential fluctuations in adherence to the exercise protocols. Moreover, we only analyzed female participants, which may limit the generalizability of the findings to males. Finally, confounding factors such as diet, sleep quality, recovery strategies, and general physical activity were not analyzed, all of which may concurrently contribute to the level of adaptations observed. Future research could employ randomized controlled trials with larger sample sizes to enhance generalizability and confirm causality. Moreover, investigating the underlying mechanisms behind JIT-induced improvements, such as mitochondrial biogenesis and muscle fiber adaptations, would provide valuable insights into its physiological effects. Longitudinal studies examining the sustainability of these adaptations beyond 6 months could further elucidate the durability of JIT’s benefits.

For dance professors and coaches, our study’s findings on JIT present practical implications for enhancing warm-up routines and improving performance outcomes among dancers. Integrating brief sessions of JIT into warm-ups has the potential to enhance aerobic capacity and muscular strength in medium term, which are essential for dance movements that demand explosive power. By incorporating JIT, instructors can target improvements in dancers’ aerobic fitness, and overall muscular performance—qualities that are important for executing dynamic and powerful dance routines. For example, coaches can supplement their regular warm-ups (2–3 times a week) with a brief session of three sets of 30-s bilateral squat jumps at a pace of 1.0 jump per second, with 30 s of rest in between. This approach, totaling just 3 min, can promote meaningful adaptations, particularly in recreational dancers, though it may have less impact on those with higher fitness levels.

## Conclusion

In conclusion, our cohort study demonstrates that incorporating JIT into warm-up routines significantly enhances aerobic capacity, peak force, and power output compared to TWU, with significant differences emerging after 3 months of training. The JIT cohort showed sustained improvements in squat jump peak force, countermovement jump peak power, and maximal strength, particularly after 6 months. Although the study’s design limits causal inferences, the findings suggest that integrating JIT into warm-up routines can effectively enhance both aerobic fitness and muscular performance, providing valuable practical strategies for dancers.

## Data Availability

The raw data supporting the conclusions of this article will be made available by the authors, without undue reservation.
